# Diagnostic and prognostic value of dual-point amyloid PET in Alzheimer’s disease (AD) mimickers

**DOI:** 10.1007/s00259-024-06676-z

**Published:** 2024-03-14

**Authors:** Luca Sofia, Federico Massa, Stefano Raffa, Matteo Pardini, Dario Arnaldi, Matteo Bauckneht, Silvia Morbelli

**Affiliations:** 1 Department of Health Science (DISSAL), University of Genoa, Genoa, Italy; 2 IRCCS Ospedale Policlinico S. Martino, Genoa, Italy; 3 Department of Neuroscience, Rehabilitation, Ophthalmology, Genetics, Maternal and Child Health (DINOGMI), Clinical Neurology, University of Genoa, Genoa, Italy; 4 Department of Medical Sciences, University of Turin, Turin, Italy; 5 Nuclear Medicine Unit, Citta’ della Salute e della Scienza di Torino, Turin, Italy


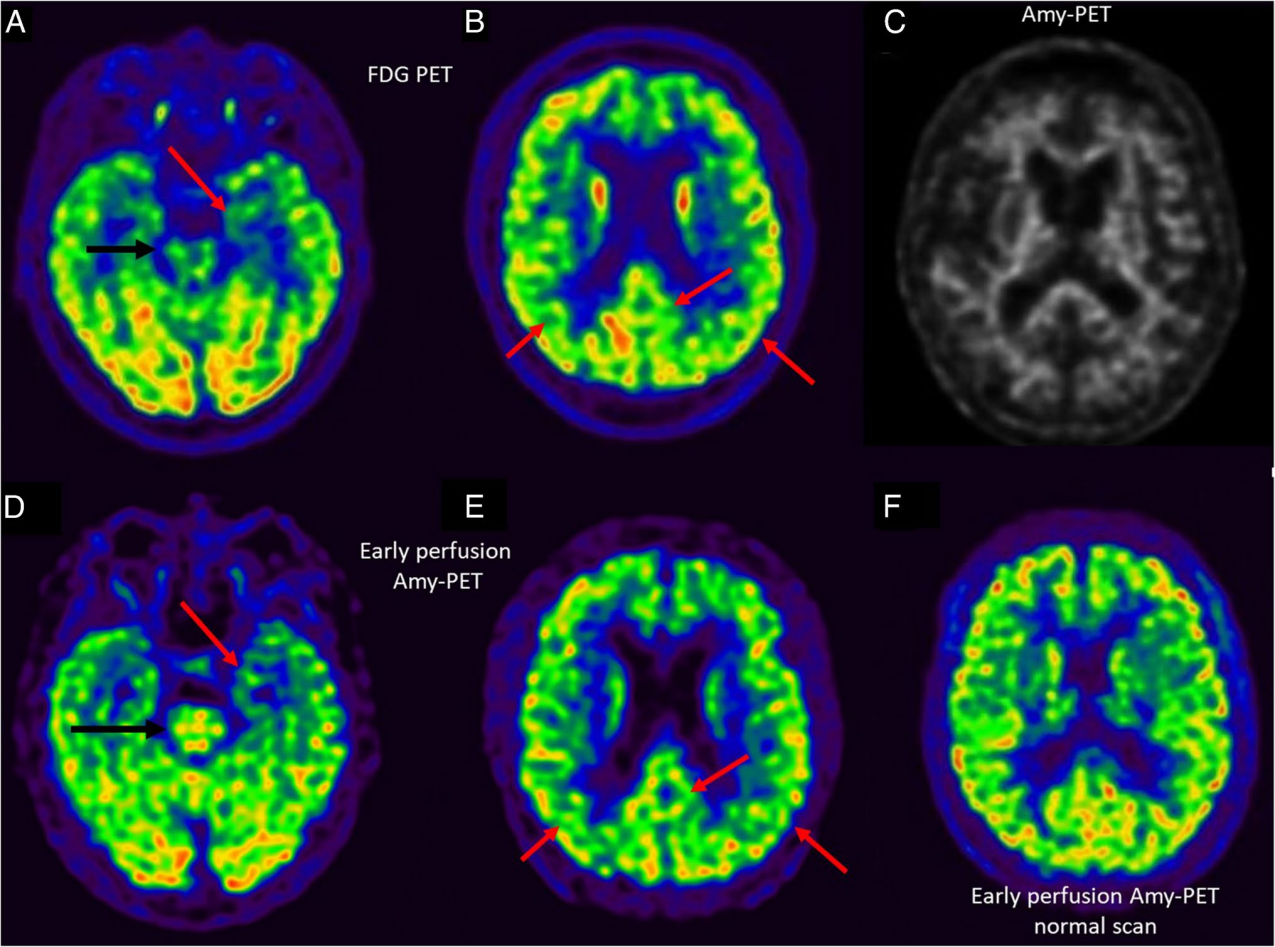


A 74-year-old man was referred to the neurologist for memory complaint. As prodromal AD was suspected, the patient underwent cerebrospinal fluid (CSF) biomarker evaluation which was not consistent with AD (negative for brain amyloidosis while mildly positive for both tau pathology and neurodegeneration: A-T + N +) [1]. To investigate non-AD underlying etiologies, the patient underwent a [^18^F]FDG-PET showing bilateral hypometabolism in the medial temporal lobes (MTL), posterior parietal cortex, and left posterior cingulate and precuneus (A, B). In the absence of brain amyloidosis, more prominent MTL hypometabolism suggested a preliminary categorization as suspected non-AD pathophysiology (SNAP) [2]. SNAP is a biomarker-based classification referring to individuals suggestive for AD-like neurodegeneration without β-amyloidosis. Tau pathology, as in primary age-related tauopathy (PART), has been hypothesized to play a major role in SNAP patients and was considered a possible etiology in this case [3]. However, the concomitant occurrence of hypometabolism in the posterior cortical regions, despite being previously described in SNAP patients due to disconnection from the hippocampus, could not allow exclusion of AD [2, 3]. Therefore, the patient underwent an amyloid-PET with [^18^F]Florbetaben which confirmed to be negative (A—according to the ATN system) (upper panel image C). Notably, amyloid-PET was acquired with a dual-point protocol including a short (5 min) image immediately after injection followed by a late steady-state standard acquisition [4]. Early acquisition is considered a perfusion-weighted phase representing a surrogate for neurodegeneration [4]. Early-perfusion amyloid PET imaging tightly mirrored the [^18^F]FDG-PET pattern (D, E; normal scan as a reference: F) [5]. Red arrows show regions of overlapping hypometabolism between [^18^F]FDG-PET and early-perfusion amyloid PET. Black arrow highlights the expected higher signal in brainstem on early-perfusion amyloid PET with respect to [^18^F]FDG. This case emphasizes the added value of dual-point amyloid-PET for the identification of AD mimickers with early perfusion imaging possibly replacing [^18^F]FDG-PET and providing prognostic stratification based on the extension/severity of neurodegeneration (although semiquantitive approaches to early-perfusion PET still need proper validation) [5]. Concomitant standard–late amyloid PET contributes to the exclusion of AD. Correlation between A/N status and topography of neurodegeneration might be relevant for diagnosis and prognosis also in AD mimickers.

## Data Availability

Data are available upon reasonable request. For related content on neuroimaging for Alzheimer’s disease, please visit: https://neuroimaging-alzheimers-disease-ime.springermedicine.com/.
